# AutoGrow4: an open-source genetic algorithm for de novo drug design and lead optimization

**DOI:** 10.1186/s13321-020-00429-4

**Published:** 2020-04-17

**Authors:** Jacob O. Spiegel, Jacob D. Durrant

**Affiliations:** Department of Biological Sciences, University of Pittsburgh, 4200 Fifth Ave, Pittsburgh, PA 15260 USA

**Keywords:** Autogrow, Genetic algorithm, Computer-aided drug design, Virtual screening, PARP-1

## Abstract

We here present AutoGrow4, an open-source program for semi-automated computer-aided drug discovery. AutoGrow4 uses a genetic algorithm to evolve predicted ligands on demand and so is not limited to a virtual library of pre-enumerated compounds. It is a useful tool for generating entirely novel drug-like molecules and for optimizing preexisting ligands. By leveraging recent computational and cheminformatics advancements, AutoGrow4 is faster, more stable, and more modular than previous versions. It implements new docking-program compatibility, chemical filters, multithreading options, and selection methods to support a wide range of user needs. To illustrate both de novo design and lead optimization, we here apply AutoGrow4 to the catalytic domain of poly(ADP-ribose) polymerase 1 (PARP-1), a well characterized DNA-damage-recognition protein. AutoGrow4 produces drug-like compounds with better predicted binding affinities than FDA-approved PARP-1 inhibitors (positive controls). The predicted binding modes of the AutoGrow4 compounds mimic those of the known inhibitors, even when AutoGrow4 is seeded with random small molecules. AutoGrow4 is available under the terms of the Apache License, Version 2.0. A copy can be downloaded free of charge from http://durrantlab.com/autogrow4.
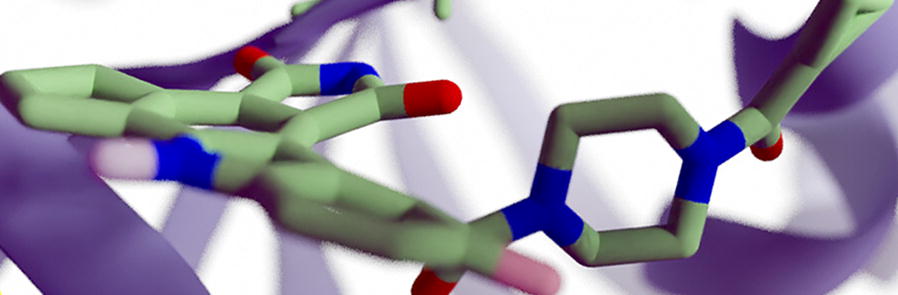

## Introduction

Computer-aided drug discovery (CADD), a critical component of many pharmaceutical pipelines, is a powerful tool that complements the expertise of medicinal chemists and biologists. Given that there are 10^20^–10^23^ synthesizable drug-like molecules [1–3], no method—experimental or computational—can hope to explore even a small subset of drug space. But CADD enables in silico experiments at scales much larger than are possible ex silico [1–5] and so can prioritize which candidate ligands warrant further testing in enzymatic or biophysical assays. CADD has been successfully applied to hit discovery, lead optimization, and compound synthesis [4–7].

CADD can be broadly divided into two categories: ligand-based drug design (LBDD) and structure-based drug design (SBDD) [6, 8]. To predict ligand binding, LBDD considers the physiochemical properties of known ligands without regard for the atomic structure of the target macromolecular receptor (e.g., protein). In contrast, SBDD predicts binding based on the receptor structure [6, 8]. SBDD can be further divided into screening and de novo approaches. Screening considers a finite database of pre-enumerated compounds, and de novo approaches generate new compounds in silico using algorithms that explore a wider range of chemistry space [4–6, 9].

We here describe AutoGrow4, a free Python-based open-source program for de novo SBDD CADD. AutoGrow4 uses a genetic algorithm (GA) to create new predicted ligands. It draws on an initial population of seed molecules to create a new population (i.e., a generation) of potential solutions (ligands). It then docks these compounds into a user-specified target protein and ranks each by its calculated fitness. New generations are seeded with the top-scoring molecules of the previous generation.

AutoGrow4 expands on the approach used in previous versions of the algorithm. The original AutoGrow, released in 2009, was one of the first de novo CADD programs to use fully flexible docking and was one of only a few free open-source programs for de novo CADD [4, 5]. More recent advances in docking software, cheminformatics libraries, and multithreading approaches [10, 11] have now enabled further improvements. AutoGrow4 has an entirely rewritten codebase that is designed to be faster, more stable, and more modular than previous versions.

To demonstrate utility, we show how AutoGrow4 can be used both to design entirely novel drug-like molecules and to optimize preexisting inhibitors. In both cases, we apply AutoGrow4 to poly(ADP-ribose) polymerase 1 (PARP-1), a well-characterized DNA-damage recognition protein. We chose PARP-1 because (1) the many known PARP-1 inhibitors (PARPi) serve as positive controls and leads to optimize [12–15]; (2) PARPi are effective treatments for many cancers with defects in the *Breast Cancer (BRCA) 1* and *2* genes [16, 17]; and (3) the PARP-1 catalytic domain has a well-characterized druggable pocket [18–20].

AutoGrow4 will be a useful tool for the CADD community. We release it under the terms of the Apache License, Version 2.0. A copy can be downloaded free of charge from http://durrantlab.com/autogrow4.

## Methods

### AutoGrow4 design and implementation

**Fig. 1:**
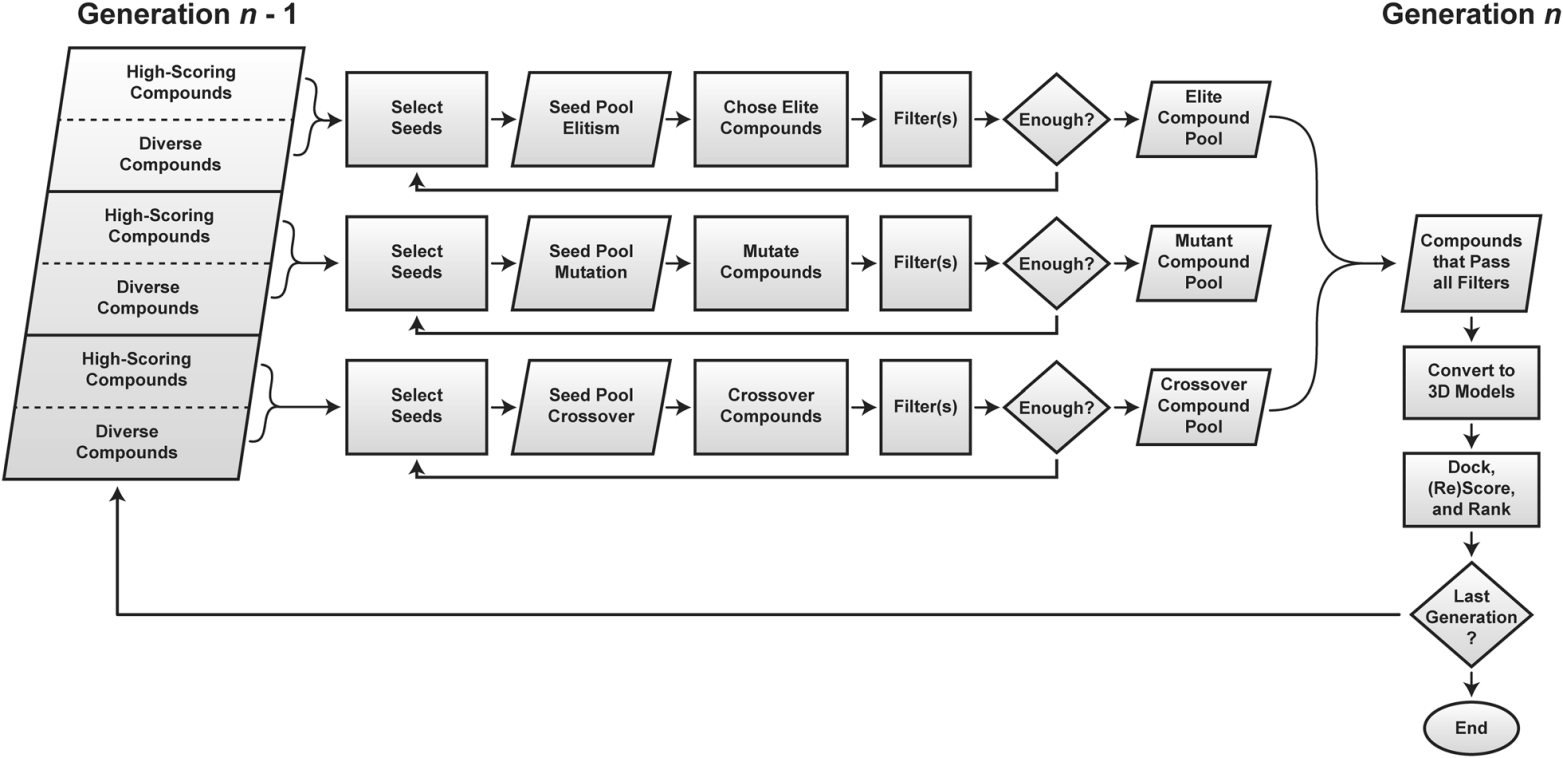
A process-flow diagram of the AutoGrow4 algorithm. Three independent seed pools are formed from the high-scoring and diverse compounds of generation *n* - 1. These are used to create the next generation of compounds (*n*) via elitism, mutation, and crossover. Compounds are converted to 3D using Gypsum-DL, converted to a dockable file format (e.g., PDBQT), docked, (re)scored, and ranked according to the fitness function

AutoGrow4 starts with an initial (input) population of compounds. This source population, called generation 0, consists of a set of chemically diverse molecular fragments (for de novo design) or known ligands (for lead optimization). AutoGrow4 creates the first generation by applying three operations to the source population: elitism, mutation, and crossover (Fig. 1). Subsequent generations are created similarly from the compounds of the immediately preceding generation.

#### Population generation via elitism

The elitism operator progresses a sub-population of the fittest compounds from one generation to the next without alterations. The AutoGrow4 elitism operator is similar to that of previous AutoGrow implementations, with two notable improvements. First, users can now optionally choose to regenerate and redock elite compounds. Many docking programs (e.g., AutoDock Vina [21]) are stochastic, so users may get slightly different poses each time. Reassessment may identify better poses than previous attempts.

Second, AutoGrow4 decouples the elitism operator from seed-population selection. In previous AutoGrow versions, the compounds that advanced via elitism were the same compounds used to seed the mutation and crossover operators (see below). With these two processes now decoupled, the user can independently control the number of compounds that advance via elitism vs. other operators.

#### Population generation via mutation

**Fig. 2:**
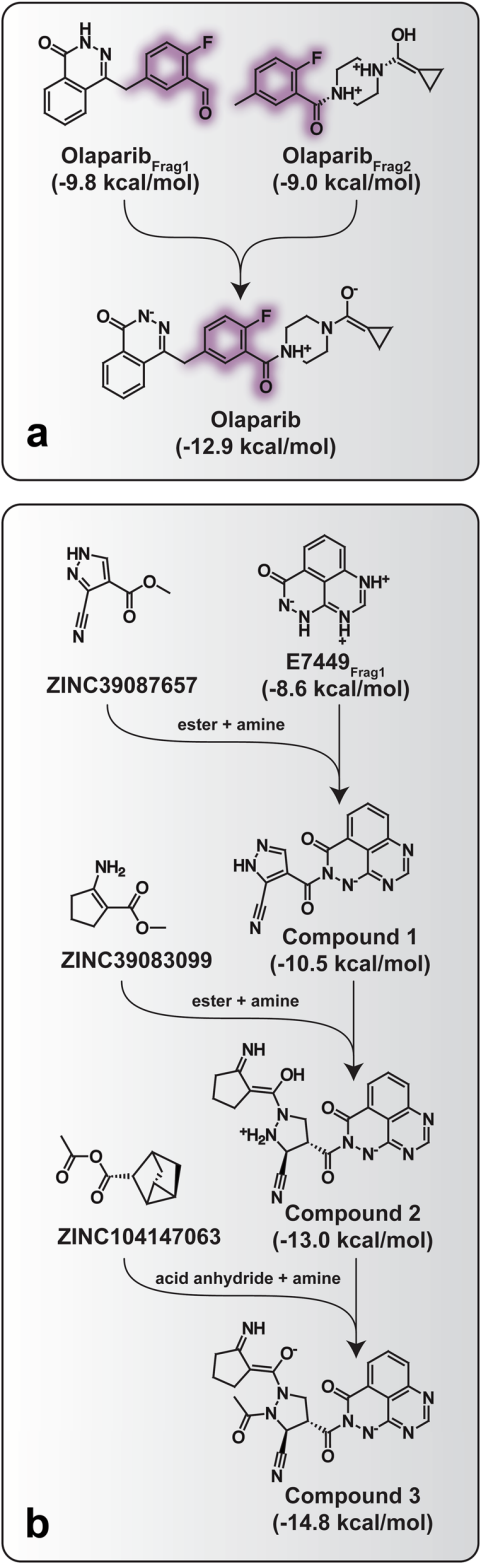
Compound lineages from a lead-optimization run seeded with PARPi fragments, chosen to illustrate crossover and mutation operations. QVina2 was used for docking. To simplify presentation, we omit some intermediate steps such as changes in protonation states.** a** Olaparib recreated via crossover from two source fragments. The largest commonly shared substructure is highlighted in purple.** b** A high-scoring compound derived from a E7449 (known inhibitor) fragment illustrates the mutation operator

The mutation operator performs an in silico chemical reaction to generate an altered child compound derived from a parent (Fig. 2b). AutoGrow4’s file-naming scheme allows the user to easily trace any mutant compound’s lineage.

The AutoGrow4 mutation operator is improved over previous versions of AutoGrow. AutoGrow 3.1.3 used AutoClickChem [22] to perform in silico reactions on 3D compound models, which required an extensive custom codebase to perform substructure searches, molecular alignments, and in silico reactions [5, 22]. In contrast, AutoGrow4 uses SMARTS-reaction notation, together with RDKit, to perform chemical mutations much faster.

This improved approach allowed us to easily add new reaction sets. Aside from the 36 click-chemistry reactions that were already possible with AutoClickChem (the AutoClickChemRxn set), AutoGrow4 also draws on a second library of 58 reactions published by Hartenfeller et al. (the RobustRxn set) [23]. We merged these two sets to form a third with 94 reactions (the AllRxn set). All sets were manually inspected, extensively unit tested, and adjusted where chemical modifications were necessary. The tutorial included with the AutoGrow4 download describes how users can create and incorporate their own custom reaction sets.

**Table 1:** The default AutoGrow4 molecular filters

Name	logP	HD; HA	MW	MR	Atoms	RotB	R	N; O; X	PSA	Sub
Lipinski [72]	$\le$ 5.0	$\le$ 5; $\le$ 10	$\le$ 500							
Lipinski* [5]	$\le$ 5.0	$\le$ 5; $\le$ 10	$\le$ 500							
Ghose [54]	-0.4 to 5.6		160–480	40–130	20–70					
Ghose* [5]	-0.4 to 5.6		160–500	40–130	20–70					
VandeWaterbeemd [107]			< 450						< 90	
Mozziconacci [108]						$\le$ 15	$\le$ 6	$\ge$ 1; $\ge$ 1; $\le$ 7		
BRENK [56]										+
NIH [109, 110]										+
PAINS [111]										+

Lipinski allows for one violation. Lipinski* is a stricter version that allows for no violations. Ghose* is a more lenient version of Ghose that allows compounds with molecular weights up to 500 Da

*HD* hydrogen-bond donor;* HA* hydrogen-bond acceptor;* MW* molecular weight (Da);* MR* molar refractivity (m^3^ mol^-1^);* Atoms* atom count;* RotB* rotatable bonds;* R* rings;* N, O, and X* nitrogen, oxygen, and halogen atoms, respectively;* PSA* polar surface area (Å^2^);* Sub* substructure searching

Seventy-nine of AutoGrow4’s 94 default reactions require two reactants. In these cases, one of the reactants is taken from a previous generation, and the other is taken from one of AutoGrow4’s complementary molecular-fragment libraries. To create these libraries, we downloaded a set of 19,274,338 commercially available small molecules from the Zinc15 database [24] on December 19, 2019. Each compound had a molecular weight (MW) less than 250 Da and a predicted octanol–water partition coefficient (logP) less than 5.0. We further filtered the compounds using the Lipinski* filter, which allows for no violations (Table 1). To ensure that all compounds could participate in at least one of AutoGrow4’s default in silico reactions, we discarded those molecules that did not possess any appropriate functional groups. We then divided the remaining compounds into functional-group categories. For each category, we retained at most the 5000 compounds with the lowest MW. These complementary libraries are included in the AutoGrow4 download in the SMILES (SMI) format. A customization option also allows users to provide their own complementary molecule libraries.

#### Population generation via crossover

The crossover operator merges two compounds from previous generations into a new compound. Like the previous version of AutoGrow (3.1.3) [5], the AutoGrow4 crossover operator finds the largest substructure that the two parent compounds share and generates a child by randomly combining their decorating moieties (Fig. 2a). AutoGrow4 embeds information about the lineage of each crossover in the compound file name, allowing users to easily examine any compound’s evolution.

AutoGrow 3.1.3 used LigMerge [25] to perform crossovers. LigMerge requires computationally expensive geometric calculations to merge 3D molecular models. In contrast, AutoGrow4 uses the RDKit Python library [26] to generate child compounds from SMILES strings of the parents. This change dramatically reduces the computational cost of compound generation and greatly simplifies the AutoGrow4 codebase.

#### Molecular filtration

AutoGrow4 uses common molecular filters to remove generated compounds with undesirable physical and chemical properties (e.g., poor predicted solubility, high biological reactivity, etc.). These compounds are eliminated before docking to avoid wasting computational resources (Fig. 1). If too few compounds pass the user-specified filter(s), AutoGrow4 automatically returns to the mutation and crossover operators to generate more candidate molecules (Fig. 1).

AutoGrow4 includes the nine predefined molecular filters shown in Table 1. Users can combine any of these filters in series. The new modular codebase also makes it easy for users to add their own custom filters that assess other molecular properties.

#### Conversion of SMILES to 3D PDB

AutoGrow4 uses the open-source program Gypsum-DL [11] to convert the SMILES representations of all new molecules into 3D models for docking. For each input SMILES, Gypsum-DL generates one or more 3D models with alternate ionization, tautomeric, chiral, cis/trans isomeric, and ring-conformational forms [11, 27]. The user can specify the pH range to use for protonation as well as the maximum number of molecular forms (variants) that Gypsum-DL should produce per input SMILES.

#### Assessing fitness

AutoGrow4 uses two metrics to assess the fitness of each solution. These fitness scores are used to select seeds for the next generation (see below). The primary fitness metric assesses how well each compound is predicted to bind the target receptor. By default, AutoGrow4 uses the docking score of the top-scoring pose. AutoGrow4 has broader docking-program support than previous versions. It supports not only AutoDock Vina [21] but also QuickVina 2.1 (QVina2) [10], a Vina-based program that runs about twofold faster. AutoGrow4 can also rescore docked poses using the NNScore1 and NNScore2 scoring functions [28, 29]. The score associated with each compound can optionally be divided by the number of non-hydrogen ligand atoms, a metric called ligand efficiency [30]. AutoGrow4’s modular, plugin-based architecture also enables long-term expandability and user customization. With the appropriate plugin code, virtually any method for docking and/or reassessing ligand binding can be integrated into AutoGrow4, including quantitative structure–activity relationship (QSAR) approaches, ligand-similarity evaluations, etc.

Aside from this primary fitness metric, AutoGrow4 also calculates a secondary fitness metric called the diversity score. The diversity score is optionally used to select seed compounds that are structurally unique compared to those of the previous generation. By seeding a new generation with a population comprised of molecules separately selected for target binding and molecular diversity, AutoGrow4 delays population convergence while still refining for the desired binding affinity.

To determine the diversity score, AutoGrow4 uses RDKit [26, 31, 32] to calculate the Morgan fingerprint of each population compound after successful docking. The similarity *s* of two compounds *mol*_A_ and *mol*_B_ is given by

1 $$\begin{aligned} s(F_A, F_B) = \frac{2 \left| F_A \cap F_B \right| }{ \left| F_A \right| + \left| F_B \right| } \end{aligned}$$

where *F*_A_ and *F*_B_ are the fingerprint bit-strings of *mol*_A_ and *mol*_B_, respectively. The value of *s* ranges from 0.0 (completely different) to 1.0 (perfectly matched). The diversity score *d* of a given molecule *mol*_M_ measures its uniqueness relative to the other molecules in its generation. The score is calculated by

2 $$\begin{aligned} d(mol_M)=\sum _{N \ne M}^{n} s(F_M, F_N) \end{aligned}$$

where the summation is over all *mol*_N_ within the generation except *mol*_M_.

#### Compound ranking and seed selection

AutoGrow4 implements Ranking, Roulette, and Tournament selection strategies [33] to choose which compounds will seed the next generation. Each strategy has its advantages and disadvantages. A Ranking selector simply chooses the best-scoring solutions. It quickly identifies local optima but often produces inbred populations of highly similar compounds [33]. In extreme cases, population convergence can cause AutoGrow4 to perpetually recreate very similar compounds each generation, without substantial improvement in fitness. A Roulette selector assigns each solution to a metaphorical roulette wheel, where the size of each area is weighted by fitness. By incorporating randomness into each generation, Roulette selection reduces the chances of becoming trapped in local optimum. But it gives all potential solutions—even the most unfit—an opportunity to advance [33]. Lastly, a Tournament selector randomly chooses a subpopulation of solutions and then selects the fittest solutions from that subpopulation. A Tournament selector incorporates more randomness than a Ranking selector while reducing the risk of selecting unfit solutions [33].

AutoGrow4 performs three subselections per generation, one for elitism, mutation, and crossover operations, respectively (Fig. 1). When using a stochastic (i.e., Roulette or Tournament) selector, these subselections are independent (i.e., each subselection picks a different but potentially overlapping set of compounds). When using a deterministic (i.e., Ranking) selector, the three subselections are identical. In all cases, each subselection separately selects compounds based on their primary (binding) and/or secondary (diversity) scores. The user can also specify the number of compounds selected based on each score type.

### Benchmark AutoGrow runs

#### Protein preparation

We tested AutoGrow4 against the PARP-1 catalytic domain. We first obtained the 4R6E structure from the Protein Data Bank (PDB) [18, 34] and removed all atoms but those of the chain A protein (4R6E:A). We used the PDB2PQR server (2.1.1, default settings) [35, 36] to add hydrogen atoms and optimize the hydrogen-bond network (pH 7). We then converted the resulting PQR file back to the PDB format using OpenBabel (2.3.1) [37].

To define the location of the binding pocket, we selected five protein residues that flank the bound, crystallographic niraparib ligand: E763, I872, G888, T907, and E988. We used the Scoria Python library [38] to calculate a bounding box that encompasses these residues. We expanded the width, length, and height of this box by a few Å to ensure that it entirely surrounded the pocket. The docking box ultimately had dimensions 25.0 Å × 16.0 Å × 25.0 Å, centered on the binding pocket. We used this PARP-1 PDB file and docking box for all AutoGrow runs (see published tutorial).

#### Comparison benchmark runs

AutoGrow 3.1.3 provides a set of 117 PDB-formatted small molecules with naphthalene substructures as source compounds [5]. To compare AutoGrow4 and AutoGrow 3.1.3, we converted these compounds to SMILES using OpenBabel 2.3.1 [37] and RDKit [26]. They are included as an SMI file in the AutoGrow4 download.

We ran the AutoGrow4 and AutoGrow 3.1.3 benchmarks on the same hardware: 12-core Xeon E5-2643v4 3.40 GHz Broadwell nodes with 512 GB RAM, provided by the University of Pittsburgh’s Center for Research Computing (CRC). We also closely matched the AutoGrow4 and AutoGrow 3.1.3 settings in terms of processor counts, population sizes, mutation reaction sets, seed molecules, and population-size/seed-size ratios. In all cases, we subjected the evolving molecules to the Ghose* and Lipinski* filters (Table 1). The complete settings are provided in Additional file 1: JSON 1 and 2.

AutoGrow 3.1.3 has some notable limitations, requiring several additional considerations. AutoGrow 3.1.3 is not Python 3 compatible, so we ran these benchmarks in a Python 2.7 environment. We also limited AutoGrow4 to one molecular variant per input SMILES, Vina 1.1.2 docking, the Ranking selector, and symmetric multiprocessing (SMP) because AutoGrow 3.1.3 does not consider alternate molecular forms, cannot use QVina2, does not implement the Roulette or Tournament selectors, and does not support message passing interface (MPI) multiprocessing [5].

All benchmark AutoGrow4 and AutoGrow 3.1.3 runs were repeated independently 24 times.

#### Large-scale de novo PARPi run

We generated a sizable, chemically diverse source library for general use when performing de novo AutoGrow4 runs. We started with the same large set of Lipinski*-filtered Zinc15 molecules described above, which was previously used to generate the default complementary small-molecule libraries required for the mutation operator. We discarded those compounds that lacked functional groups capable of participating in at least one of AutoGrow4’s 94 default reactions. The remaining compounds were grouped by MW ($\le$ 100 Da, 100 Da < MW $\le$ 150 Da, 150 Da < MW $\le$ 200 Da, and 200 Da < MW $\le$ 250 Da). To keep the source library reasonably sized while maintaining chemical diversity, we randomly discarded excess compounds in each MW category that had overrepresented functional groups. Ultimately, at most 100 compounds remained for each functional group in each weight range. These four source-library sets, which collectively comprise 24,595 molecules, are included in the AutoGrow4 download.

The large-scale de novo run described in "Results and discussion" was seeded with small molecules from the 100 Da < MW $\le$ 150 Da category. This run used MPI multiprocessing, QVina2 docking, and the Ranking selector in a Python 3.7 environment. It ran for thirty generations on ten MPI-enabled CRC computer nodes with 28-core Broadwell Processors and 64 GB RAM/node, networked with Intel’s Omni-Path communication architecture. Evolving molecules were subjected to the Ghose, Lipinski*, and PAINS filters (Table 1). In the first generation, AutoGrow4 generated 40, 500, and 500 compounds via elitism, mutation, and crossover, respectively. In subsequent generations, it generated 500, 2500, and 2500 compounds via elitism, mutation, and crossover, respectively. The complete settings are provided in Additional file 1: JSON 3. We used BlendMol [39] to generate figures of representative docked molecules.

#### PARPi lead-optimization runs

To show how AutoGrow4 can be used for lead optimization, we generated a focused source library of 94 seed molecules. This library includes eleven known PARPi, identified using http://www.clinicaltrials.gov. It also includes 83 PARPi molecular fragments derived from those eleven via Breaking of Retrosynthetically Interesting Chemical Substructures (BRICS) decomposition [7].

We used AutoGrow4 with this small source library to predict new PARP-1 ligands similar to known inhibitors. To focus computational effort on the chemistry space near known PARPi, we ran AutoGrow4 for only five generations, but with demanding docking-exhaustiveness, variant-per-molecule, and population-size settings. Evolving molecules were subjected to the Ghose, Lipinski*, and PAINS filters (Table 1). The complete settings are provided in Additional file 1: JSON 4. The settings of the first generation were conservative because the small source library had a limited number of reactive functional groups. We thus limited the first generation’s size to 40, 500, and 500 molecules derived using the elitism, mutation, and crossover operators, respectively. Subsequent generations were larger. They included 250, 2500, and 2500 molecules derived using the elitism, mutation, and crossover operators, respectively.

These lead-optimization runs were repeated six independent times using Python 3.7 on the same MPI-enabled nodes available through the CRC. We again used BlendMol [39] to generate figures of representative docked molecules.

### PARPi-like compounds: AutoGrow4 optimization vs. similarity-based screening

To compare AutoGrow4 lead optimization to a more traditional similarity-based virtual screening (VS) approach [40, 41], we generated a library of small molecules that are structurally similar to known PARPi. We downloaded the structures of ~2200 known PARPi from the BindingDB database [42, 43] on March 14, 2020. Many of these compounds were close analogues, so we used a Tanimoto-based clustering algorithm [44] (Tanimoto cutoff: 0.65) to group the compounds into 40 clusters. We then selected one molecule per cluster to construct a set of structurally unique known PARPi.

For each of these PARPi, we next downloaded at most 250 compounds from PubChem [45] with corresponding Tanimoto coefficients greater than 0.80 (8444 unique molecules). To maximize structural diversity and reduce the number of compounds, we again applied the clustering algorithm (Tanimoto cutoff: 0.2), yielding a set of 4631 PARPi-like molecules that (1) were not in the original PARPi set and (2) could be docked into PARP-1 with QVina2.

## Results and discussion

### Poly(ADP-ribose) polymerase 1

We used PARP-1, a protein critical for DNA repair, as a test system to demonstrate AutoGrow4 utility. DNA is under constant threat of damage by ionizing radiation, UV radiation, and reactive oxygen species [16, 46]. Base excision repair (BER) is a crucial pathway for repairing single-strand DNA (ssDNA) breaks, and non-homologous end joining (NHEJ) and homologous recombination (HR) are critical pathways for repairing double-strand DNA (dsDNA) breaks [16, 46–49]. When PARP-1 recognizes sites of DNA damage, it begins to convert NAD+ molecules into a negatively charged network of poly(ADP-ribose) (PAR) chains [16]. These PAR chains are covalently attached to nearby proteins, including PARP-1 itself, through a process known as PARylation [16]. This PARylation signal recruits DNA repair proteins (e.g., NHEJ and BER) [16, 50].

Defects in the *BRCA 1* and *2* genes, prevalent in breast and ovarian cancers, cause loss of HR repair function [16]. HR-defective cancer cells rely heavily on NHEJ and BER to compensate, so much so that further loss of NHEJ and BER is lethal [16]. PARPi capable of disrupting BER and NHEJ signaling are thus potential therapeutics for treating HR-defective breast-cancer cells [16, 17]. In contrast, non-cancerous cells survive PARPi exposure because their HR-repair mechanisms are intact [16, 17]. HR is also most active during the S to M transition, so actively dividing tumor cells are especially vulnerable [16, 51, 52]. Four PARPi are FDA approved (olaparib, rucaparib, niraparib, and talazoparib), and several more are in clinical trials [12–15].

### Comparison Benchmark runs

**Fig. 3:**
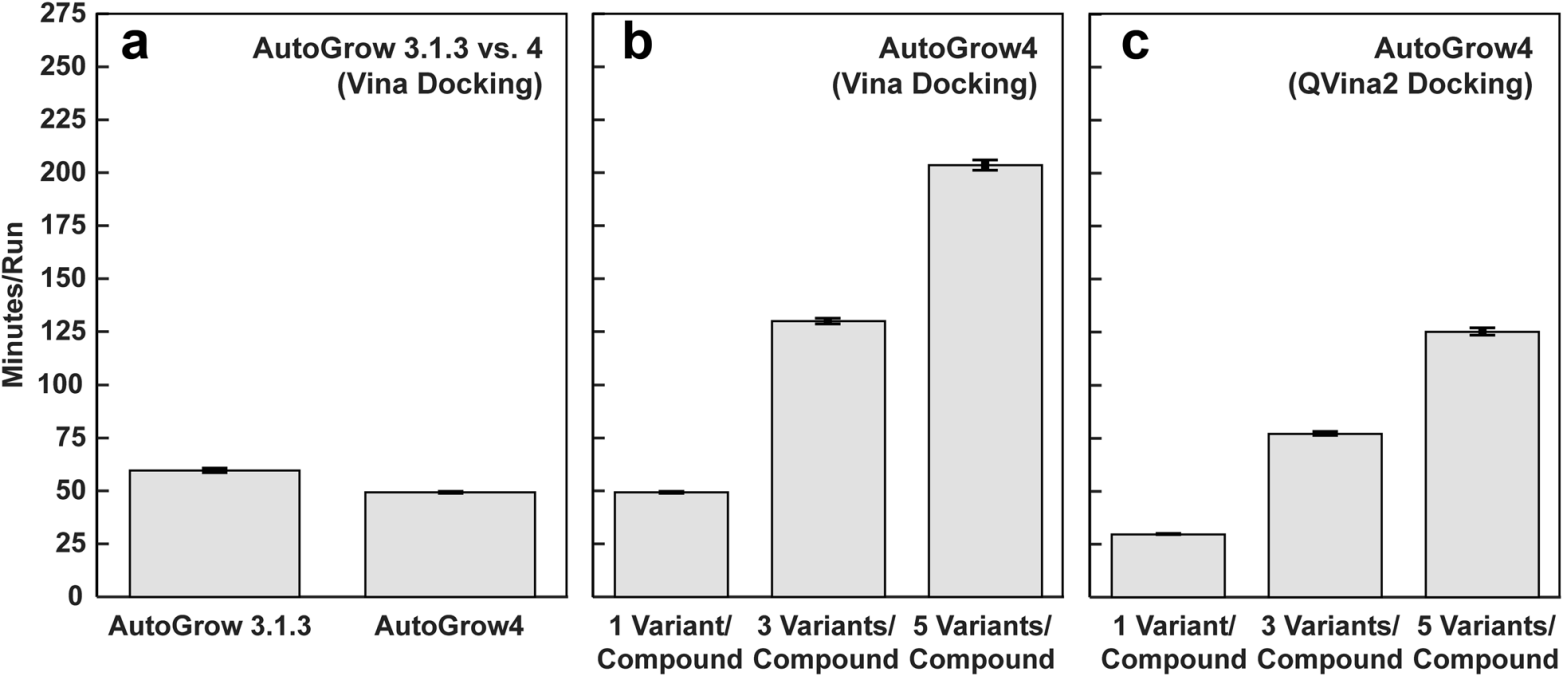
Benchmark AutoGrow runs. Bar heights show the mean times required to complete five generations, averaged over 24 runs. Error bars represent standard errors.** a** A comparison of AutoGrow 3.1.3 and AutoGrow4, when run using similar settings (see "Methods").** b** AutoGrow4 runs when generating at most one, three, and five variants per input compound, using Vina docking.** c** AutoGrow4 runs when generating at most one, three, and five variants per input compound, using QVina2 docking

To compare AutoGrow4 and its predecessor AutoGrow 3.1.3, we ran both programs 24 times using similar settings. On average, AutoGrow4 completes five generations 1.21 times faster than AutoGrow 3.1.3 (59.64 vs 49.34 min/run, see Fig. 3a).

We also compared AutoGrow4 performance under different user-parameter conditions. AutoGrow4 has been outfitted with many new features not available in previous versions. Of note, it uses Gypsum-DL [11] to generate alternate ionization, tautomeric, chiral, cis/trans isomeric, and ring-conformational variants of each input SMILES string. Figure 3b shows that AutoGrow4 run times vary roughly linearly with the specified maximum number of variants per input molecule (Additional file 1: JSON 2). It is worth noting that in practice Gypsum-DL often produces fewer variants than the maximum specified. In these benchmarks, specifying a maximum of three and five produced 2.5 and 3.9 variants per compound.

Finally, we assessed AutoGrow4 performance when docking with QVina2 (Fig. 3c). Compared to the other steps in our algorithm, docking is particularly time consuming. AutoGrow4’s ability to dock with QVina2 in addition to Vina is a notable improvement over previous versions. When set to generate at most five variants per compound, AutoGrow4 runs 1.6 times faster when docking with QVina2 vs. Vina (125.04 vs. 203.52 min/run, respectively; Fig. 3b, c).

### Large-scale de novo PARPi run

#### Predicted ligands

**Fig. 4:**
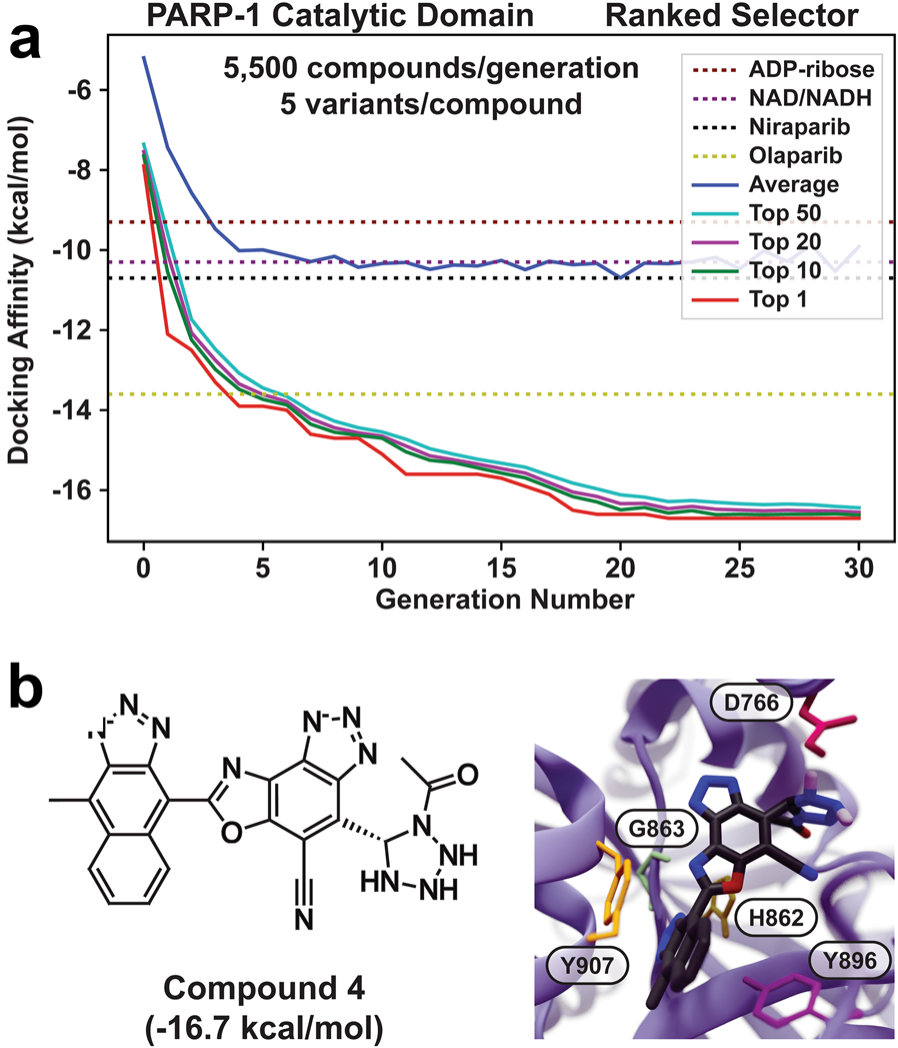
Results of a large-scale de novo run.** a** QVina2 scores per generation. The average score of all compounds per generation is shown in blue. The average scores of the top 50, 20, 10, and 1 compounds are shown in cyan, purple, green, and red, respectively. The QVina2 scores of known ligands are shown as dashed lines.** b** The AutoGrow4-generated compound with the best QVina2 score (30th generation). The PARP-1 catalytic domain was used for docking (PDB ID: 4R6E:A, shown in blue ribbon). Select protein residues are shown in colored sticks representation

We performed an extensive PARP-1 run to show how AutoGrow4’s parallelization and multiprocessing capabilities enable large-scale de novo design. These runs used a large, diverse library of seed molecules and fragments to produce high-scoring compounds such as Compound 4, which has a QVina2-predicted binding affinity of -16.7 kcal/mol (Fig. 4). This predicted ligand has a 1*H*-naphtho[2,3-*d*][1,2,3]triazole substructure that forms $\uppi$–$\uppi$ stacking interactions with the PARP-1 Y907, H862, and Y896 residues. Interestingly, two of these residues (H862 and Y896) belong to the PARP-1 catalytic triad, which is conserved in PARP-1 through PARP-6 [53]. Additional hydrogen bonds form between the compound’s cyclic nitrogen atoms and the backbone atoms of G863, R865, and R878. An electrostatic interaction with D766 is also possible, depending on the protonation states of D766 and the compound tetrazolidine substructure.

#### A caution regarding chemical properties

Though longer AutoGrow4 runs can produce compounds with remarkable scores, we generally recommend multiple independent runs with fewer generations. Longer runs have several disadvantages. First, the evolving compounds increasingly take on chemical properties that are artefactually favored by the fitness function and/or ligand-creation operations. For example, Compound 4 (Fig. 4b), one of the best-scoring compounds produced by the extensive PARP-1 de novo run, has a molecular weight (MW) near the 480 Da maximum that the applied Ghose filter permits (478.1 Da) [54]. The Vina scoring function is known to favor larger molecules [55], perhaps explaining in part this apparent evolutionary tendency towards increased MW. Filters that place tighter restrictions on MW, as well as ligand-efficiency rescoring [30], can mitigate this bias.

Second, longer runs can lead to the accumulation of undesirable moieties. The 24th-generation compound shown in Additional file 1: Figure S1, one of the highest scoring compounds from our large-scale de novo run, provides a good example. This compound possesses azo and ethyne moieties, which belong to a broad category of substructures thought to be mutagenic, pharmacokinetically unfavorable, reactive, and/or likely to interfere with typical high-throughput screening approaches [56]. This challenge, typical of longer runs, can be mitigated by applying the appropriate filter(s) (e.g., the BRENK filter [56]).

Third, compound synthesizability, also a critical chemical property, similarly tends to diminish in later generations as the accumulation of mutation and crossover events causes the population to drift from the source molecules.

#### A caution regarding homogeneity and convergence

Long runs also suffer from population homogeneity and premature convergence. In this scenario, the fitness scores of existing molecules are so good that new compounds generated via mutation and crossover can rarely outcompete them [57]. Compound fitness thus tends to improve quickly in the earliest generations but stalls in later generations despite consuming comparable computational resources. For example, the average docking score of the top 50 molecules in our large-scale de novo run improved -6.09 kcal/mol from generation zero to five (-7.36 kcal/mol to -13.45 kcal/mol, respectively), but it only improved another -2.99 kcal/mol by generation 30 (-16.44 kcal/mol). The populations began to converge by generation 20, with only minor subsequent improvements in fitness (Fig. 4a).

AutoGrow4 uses several strategies to avoid population convergence and homogeneity. First, its sizable libraries of diverse seed molecules encourage the exploration of a large subset of chemistry space, at least in early generations. Second, it considers both primary (binding) and secondary (diversity) scores when selecting molecules for elitism, mutation, and crossover operations (Fig. 1). By seeding each generation with a combination of well docked and unique compounds, AutoGrow4 aims to search more of chemistry space while still maintaining a selective pressure for reasonable predicted ligands. Finally, AutoGrow4 provides different selection strategies (e.g., Roulette and Tournament) that may delay convergence. Despite these measures, multiple independent runs of fewer generations are typically more computationally efficient.

### PARPi lead-optimization runs

#### AutoGrow4 applied to lead optimization

We performed six short PARP-1 runs to show how AutoGrow4 is a useful tool for lead optimization. These runs used 94 known PARPi and PARPi fragments as seeds rather than a large library of diverse molecular fragments. Our ultimate goal was to evolve molecules that are chemically similar to known ligands, but with improved docking scores. To perform a narrow but thorough search of the chemistry space centered around the initial leads, we ran each AutoGrow4 run for only five generations. But we used large population sizes (see "Methods") and increased the QVina2 *exhaustiveness* parameter to 25 to improve the chances of finding optimal docked poses.

By the third generation, the average QVina2 score of the top-20 compounds across all six runs (the grand mean) already matched the score of the best-scoring known PARPi, olaparib (-13.6 kcal/mol, AstraZeneca). By the fifth generation, the grand mean of the top-50 compounds (-14.0 kcal/mol) was better than the olaparib score (Additional file 1: Figure S2).

**Fig. 5:**
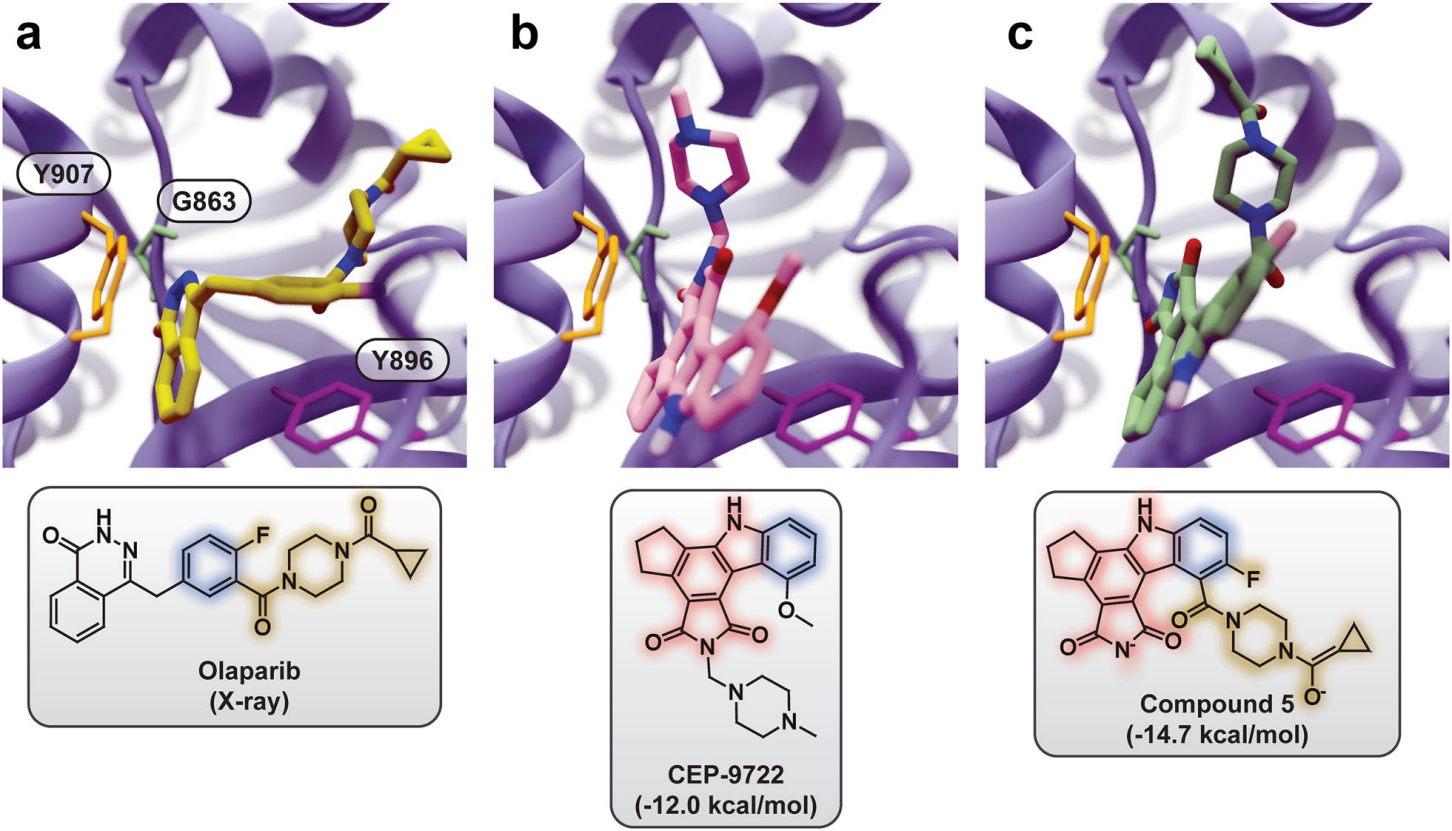
Example ligand poses and structures. Select protein residues are shown in colored sticks representation (top row). Common substructures are highlighted in blue, yellow, and pink (bottom row).** a** The crystallographic olaparib pose (PDB: 5DS3), aligned and superimposed on the 4R6E:A structure for comparison’s sake (blue ribbon).** b** CEP-9722 docked into the 4R6E:A structure.** c** Compound 5, derived from olaparib and CEP-9722 fragments, docked into the 4R6E:A structure

Of all the molecules generated during the six PARPi lead-optimization runs, compound 5 had one of the best QVina2 scores (Fig. 5 and Additional file 1: Figure S3, -14.7 kcal/mol). We focused subsequent analysis on this molecule rather than the best-scoring compound (Fig. 2b) because compound 5 was derived from two source-library PARPi fragments (Olaparib_Frag3_ and CEP-9722_Frag1_), the result of a crossover in the first generation (Additional file 1: Figure S3). It thus provides an excellent example of AutoGrow4-guided lead optimization.

Compound 5 participates in $\uppi$–$\uppi$ stacking interactions with the PARP-1 Y907 and Y896 residues. One of its carbonyl oxygen atoms also forms a hydrogen bond with G863 (Fig. 5c). These interactions are typical of the binding modes of known PARPi [18–20, 58–66] such as the crystallographic olaparib pose (Fig. 5a) [19] and a docked CEP-9722 pose (Cephalon, Fig. 5b).

In constructing compound 5, AutoGrow4 attached an olaparib-derived piperazine moiety at a different position than the CEP-9722 piperazine, but the docked poses of both compounds orient their respective piperazines similarly. Interestingly, this orientation differs from that of the olaparib piperazine (Fig. 5). We note that the crystallographic poses of several other PARPis position piperazine moieties at alternate locations [19, 66–68]. For example, the crystal structure of the potent PARPi EB_47_ [69, 70] bound to PARP16 (PDB ID: 6HXR) places a piperazine substructure near that of our compound 5 docked pose. Notably, EB_47_ was not among the source-library compounds used for the PARP-1 lead-optimization runs.

#### AutoGrow4 vs. other docking techniques for lead optimization

Similarity-based virtual screening (VS) is a popular technique for in silico ligand optimization [40, 41]. One first generates a library of compounds that are chemically similar to known ligands. VS is then used to prioritize the compounds in hopes of ultimately identifying molecules that bind with better predicted affinities than those of the known ligands. Two methods for identifying chemically similar compounds are common: substructure and similarity searching.

In the substructure scheme, the compound library consists of molecules that share substructures in common with known ligands. The generation-1 compounds of each AutoGrow4 lead-optimization run form such a library because (1) they are derived from the PARPi fragments/molecules of generation 0, and (2) both the mutation and crossover operators generate compounds that share substructures in common with parent molecules. The progress made from generation 1 to generation 5 thus illustrates how AutoGrow4 optimization can improve scores beyond substructure-based VS alone. As shown in Additional file 1: Figure S2, the grand-mean docking score of the top 50 compounds from generation 1 ($\sim$ 1% of all compounds screened that generation) was -12.3 kcal/mol. Following five generations of AutoGrow4 optimization, that grand-mean score improved to -14.0 kcal/mol.

In the similarity-searching scheme, the compound library consists of molecules that are structurally similar in their entirety to known ligands, per a whole-molecule metric such as the Tanimoto coefficient [71]. To compare this in silico optimization method to our GA approach, we generated a library of 4631 PARPi-like molecules. These compounds were processed with Gypsum-DL [11] and docked into PARP-1 with QVina2 [10] using the same parameters used in the AutoGrow4 lead-optimization runs.

The average docking score of the top-50 compounds from this similarity library ($\sim$1% of all unique compounds docked) was -12.7 kcal/mol. In contrast, five generations of AutoGrow4 optimization produced compound sets with top-50 average scores around -14.0 kcal/mol. This comparison of course has its limitations. Generating the similarity library using stricter Tanimoto cutoffs would have likely improved the average scores, though at the expense of structural diversity. In contrast, had we run the AutoGrow4 lead-optimization runs for additional generations, our GA method would have likely identified compounds with improved docking scores. But the comparison nevertheless contextualizes the AutoGrow4 approach.

### AutoGrow4 operators and molecular weight

In evaluating AutoGrow4, we also carefully studied the program’s tendency to evolve compounds with increasing MW. The AutoGrow4 mutation and crossover operators could in theory drive this tendency. On the other hand, larger ligands often form more molecular interactions with their targets, so the observed MW increases over time may reflect physiochemical reality. The Vina scoring function may also explain the tendency towards greater MW, given its known bias in favor of larger molecules [55].

To determine the role AutoGrow4 operators play in the observed tendency, we first explored the impact of the mutation operator on MW. From among all the operations performed during the six PARPi lead-optimization runs, we identified 55,683 mutation events involving an AutoGrow4-generated reactant. On average, each of these operations increased MW by 28% (66.0 Da). But in 11% of cases, the MW decreased. To illustrate how this is possible, consider the transesterification of phenyl benzoate and methanol. The resulting products (methyl benzoate and phenol) both have MWs less than the phenyl benzoate reactant.

We next explored the impact of the crossover operator on MW. We identified 50,169 crossover events from the six PARPi lead-optimization runs that involved two AutoGrow4-generated parent compounds. On average, the MW of the resulting child compound was only 5% larger (11.5 Da) than the average MW of the two parents. In 43% of cases, the MW decreased because the child molecule inherited a low-weight set of decorating moieties from the parents.

These results show that the mutation and crossover operators may promote some compound growth, but they often reduce compound size as well. Users who wish to limit AutoGrow’s tendency towards larger-MW molecules may be interested in the included molecular filters that place tighter restrictions on MW (e.g., Lipinski [72], Ghosh [54]). We also recommend source (generation 0) populations comprised of small molecular fragments to maximize the number productive AutoGrow4 generations executed before running into MW filter cutoffs. Increasing the number of crossover operations per generation may also effectively control MW, given that crossovers are more likely to reduce MW than are mutations. Finally, users can instruct AutoGrow4 to rescore docked molecules by ligand efficiency [30], which normalizes docking scores by the number of compound heavy atoms and so penalizes larger molecules.

### Identifying critical protein–ligand interactions

Beyond de novo generation and lead optimization, AutoGrow4 provides a systematic way of identifying pharmacologically important protein–ligand interactions. Cataloguing the most common interactions among top-scoring AutoGrow4-generated compounds can inform subsequent experiments ranging from QSAR drug design to site-directed mutagenesis.

**Table 2:** The protein–ligand interactions of the 100 best-docked compounds from the large-scale de novo run, per BINANA

	D766	D770	H862	G863	R865	R878	Y907
Cation-$\uppi$	0	0	98	0	0	0	0
Hydrogen bond	3	0	1	100	56	47	1
Electrostatic	100	41	3	0	0	9	0
T-stacking	0	0	71	0	0	0	0
$\uppi$–$\uppi$	0	0	100	0	0	0	100

Infrequent interactions (< 10%) are excluded. Values are given as percents

The large-scale de novo run provides many useful examples of compounds with high predicted affinities. We identified the 100 compounds with the best docking scores from among the hundreds of thousands of docking events performed over 30 generations of evolution. We then used the BINANA 1.1.2 algorithm [73] to automatically characterize the protein–ligand interactions of each best-docked pose. Four interactions were present in all 100 docked poses: two separate $\uppi$–$\uppi$ stacking interactions with Y907 and H862, an electrostatic interaction with D766, and a hydrogen-bond interaction with G863 (Table 2). Several other interactions were prevalent, though not universal: an electrostatic interaction with D770 (41%), a hydrogen-bond interaction with R865 (56%), and electrostatic and hydrogen-bond interactions with R878 (9% and 47%, respectively) (Table 2). The crystallographic poses of known PARPi (e.g., olaparib) participate in many of the same interactions seen among the top AutoGrow4 compounds [19]. Our large-scale de novo run—which was seeded with random molecular fragments not necessarily related to known PARPi—thus serves as a blind-study validation of AutoGrow4’s ability to identify pharmacologically important catalytic-pocket interactions.

Given that many of the AutoGrow4 generated compounds have better docking scores than known PARPi, the in silico compounds provide insight into future optimization strategies. For example, all 100 of the top-scoring AutoGrow4-generated compounds (large-scale de novo run) form electrostatic interactions with D766, but olaparib does not. Adding a positively charged moiety to the olaparib piperazine might enable an additional interaction with D766.

These results reinforce the critical role Y907 plays in high-affinity binding. Many known co-crystallized PARPi participate in a $\uppi$–$\uppi$ stacking interaction with Y907 [18–20, 58–66]. The top-100 compounds (per the docking score) produced in both the large-scale de novo and lead-optimization runs are all predicted to interact with Y907, suggesting that this interaction may be broadly critical regardless of the chemical scaffold. Unfortunately, interactions with Y907 also raise concerns for the future of orthosteric PARPi. When the receptor tyrosine kinase c-Met phosphorylates Y907, PARP-1 catalytic activity increases and PARPi binding affinity weakens [74]. c-Met phosphorylation thus provides a potential mechanism for PARPi resistance [74]. Consequently, there is a need for improved PARPi that do not rely on any interaction with Y907. Based on our AutoGrow4 results, we hypothesize that developing catalytic-pocket inhibitors that do not interact with Y907 will be difficult. A better strategy may be to target other (allosteric) pockets or to pursue cocktail treatments that inhibit both PARP-1 and c-Met.

### Comparison with other programs

Over the years, a number of programs have been developed to assist with de novo drug design [75–91]. A comprehensive review is beyond the scope of this article, but a few programs, summarized in Table 3, warrant specific mention. MoleGear is a recently published algorithm that also takes an evolutionary approach to de novo drug design [92]. It provides a graphical user interface and allows users to dock compounds with either AutoDock [93] or AutoDock Vina [21]. But despite its recent publication, MoleGear does not appear to be publicly available, and the program is closed source.

**Table 3:** A comparison of several de novo design programs

Program	FOSS	Docking options	MPI enabled	OS
AutoGrow4	Yes	Vina/QVina2/customizable	Yes	Linux/macOS/Windows (via Docker)
MoleGear [92]	No	Autodock and Vina	Yes	Unspecified
GANDI [96]	Yes	DAIM/SEED/FFLD	Yes	Linux
de novo DOCK [94]	Yes	DOCK	Yes	Linux/macOS
REINVENT [100]	Yes	N/A	Unspecified	Linux/macOS
LigDream [101]	Yes	N/A	Unspecified	Unspecified

FOSS stands for “free and open source software”

In contrast, de novo DOCK [94] is an open-source algorithm that is integrated into the DOCK6 docking program itself [95]. To produce novel compounds, it uses an iterative fragment-growth method that is based on the DOCK6 anchor-and-grow search algorithm [94]. The method first identifies core components of a given compound, referred to as anchors, and then expands that anchor layer by layer via fragment addition. Though de novo DOCK is a powerful program, AutoGrow4 has several advantages. First, AutoGrow4 is not tied to a specific docking program. Users can choose between Vina and QVina2 docking by default, and AutoGrow4’s plugin-based architecture makes it easy to incorporate other docking programs as well. Second, AutoGrow4 uses high-yielding in silico chemical reactions to generate compounds via mutation. In contrast, de novo DOCK does not provide a reaction-based mutation operator.

The free and open-source program GANDI takes a different approach to de novo design [96]. It first docks and scores molecular fragments using the DAIM [97], SEED [98], and FFLD [99] programs. It then joins promising fragments via a molecular linker taken from a predefined look-up table. GANDI uses a GA that employs a parallel-model approach, often referred to as an island model [96]. It evolves multiple populations separately, only occasionally swapping molecules between them. GANDI’s fragment-and-linker approach, though effective, does limit the search space to compounds that can be generated using a pre-defined set of linkers. In contrast, AutoGrow4 effectively allows any linker regions to evolve with the rest of the compound.

Recent efforts have also used machine learning for de novo design. For example, the open-source program REINVENT [100] uses recurrent neural networks and reinforcement learning to generate de novo compounds. LigDream, another example, uses a convolutional neural network [101] and focuses training instead on the 3D shapes of known ligands. Machine-learning approaches such as these are effective, but they must often be trained on preexisting ligands. In contrast, AutoGrow4 can generate compounds in the absence of known inhibitors (see the large-scale de novo run above).

## Conclusions

AutoGrow4 is a powerful program for hit discovery and lead optimization, particularly when paired with expert knowledge about the target pocket and any known ligands. We view AutoGrow4 as an open-source tool for hypothesis generation. It effectively narrows the vast scope of all possible compounds to a subset of candidate ligands. Expert users must then apply their own biological and chemical understanding to properly interpret the results and to ensure that the generated compounds are chemically feasible.

AutoGrow4 is available free of charge under the terms of the open-source Apache License, version 2.0. A copy of the latest version can be downloaded from http://durrantlab.com/autogrow4, and an archived copy is provided as Additional file 2. AutoGrow4 is compatible with both Python 2.7 and 3.7. Users must separately install the following third-party Python-library dependencies: RDKit [26], numpy [102, 103], scipy [104], matplotlib [105], and func_timeout (available via *pip*). If the mpi4py Python package is installed [106], AutoGrow4 can leverage multiple processors using MPI on MPI-enabled clusters. Finally, to convert structures from the PDB to the PDBQT format for use with Vina and QVina2, AutoGrow4 requires either AutoDock MGLTools or Open Babel [37, 93].

Installation instructions for AutoGrow4 and its dependencies are provided in the AutoGrow4 tutorial. AutoGrow4 runs on Linux and macOS. We strongly encourage use of the AutoGrow4 Docker container (Docker, Inc.), included in the download, which automatically installs all dependencies and further enables use on Windows.

## Supplementary information

**Additional file 1.** The Additional file includes detailed descriptions of the AutoGrow 3.1.3 and AutoGrow4 parameters used in the benchmark and PARP-1 runs. It also includes Figures S1, S2, and S3, referenced in the text.

**Additional file 2.** An archive of the AutoGrow4 source code. See http://durrantlab.com/autogrow4 for the latest version.

## Data Availability

All data and materials used to validate AutoGrow4 are included in the AutoGrow4 download.
