# Supplementary material for: AutoGrow4: an open-source genetic algorithm for de novo drug design and lead optimization
Source: J Cheminform. 2020 Apr 17;12:25. doi: 10.1186/s13321-020-00429-4 (PMC7165399; doi:10.1186/s13321-020-00429-4)
Supplement: Supplementary file 2 — Additional file 2. An archive of the AutoGrow4 source code. See http://durrantlab.com/autogrow4 for the latest version. [file 13321_2020_429_MOESM2_ESM.pdf]

Supplemental Information

Supplemental JSON Files:

Supplemental JSON 1: Benchmark Settings for AutoGrow 3.1.3 2

Supplemental JSON 2: Benchmark Settings for AutoGrow4 3

Supplemental JSON 3: Settings for AutoGrow4 Large-Scale *de novo* Run 4

Supplemental JSON 4: Settings for AutoGrow4 PARPi Lead-Optimization Run 5

Supplemental Figures:

Figure S1: A High-Scoring Compound Predicted to Bind the PARP-1 Catalytic Domain 6

Figure S2: Results of Six Independent PARPi Lead-Optimization Experiments 7

Figure S3: The Lineage of Compound 5, Created in the First Generation of a PARPi Lead-Optimization Run 8

```
{
        "filename_of_receptor": "/autogrow4/tutorial/PARP/4r6eA_PARP1_prepared.pdb",
        "center_x": -70.76,
        "center_y": 21.82,
        "center_z": 28.33,
        "size_x": 25.0,
        "size_y": 16.0,
        "size_z": 25.0,
        "additional_autoclickchem_parameters": "+all_reactions”
        "allow_modification_without_frag_addition": true,
        "directory_of_source_compounds":
        "/autogrow/autogrow/tutorial/starting_compounds/",
        "directory_of_fragments": "/autogrow/autogrow/fragments/MW_150/",
        "number_of_mutants_first_generation": 50,
        "number_of_crossovers_first_generation": 50,
        "number_of_mutants": 85,
        "number_of_crossovers": 85,
        "top_ones_to_advance_to_next_generation": 70,
        "num_generations": 6,
        "max_seconds_per_generation": 18000,
        "use_lipinski_filter": true,
        "use_strict_lipinski_filter": true,
        "use_ghose_filter": true,
        "scoring_function": VINA,
        "score_by_ligand_efficiency": false,
        "maintain_core": false,
        "minimum_core_atoms_required": 4,
        "vina_executable":
        "/autogrow4/autogrow/docking/docking_executables/vina/autodock_vina_1_1_2_linux
        _x86/bin/vina",
        "num_processors": 12
}
```


Supplemental JSON 1: AutoGrow 3.1.3 settings used in the benchmark experiments. Settings are presented in JSON format for comparison's sake, though AutoGrow 3.1.3 does not accept JSON format. Any unspecified settings were set to the AutoGrow 3.1.3 defaults.

In running these benchmarks, we compensated for several differences between AutoGrow 3.1.3 and AutoGrow4. In all generations but the first, AutoGrow 3.1.3 treats compounds that advance via elitism as new compounds. In contrast, AutoGrow4 does not count elite compounds against the total to create. To make as fair a comparison as possible, we increased the *number_of_mutants* and *number_of_crossovers* to compensate. This adjustment ensured that AutoGrow 3.1.3 would create exactly 100 new compounds in each generation, separate from the 70 compounds that advanced via elitism. AutoGrow 3.1.3 also produces an extra generation that summarizes the best compounds from all previous generations. This extra generation neither creates nor tests any compounds, but simply handles input/output operations. We thus set the *num_generations* parameter to six generations to produce five production generations.

Paths beginning with */autogrow/*, such as the directory of source compounds, refer to files contained within the AutoGrow 3.1.3 download. Paths beginning with /*autogrow4*/, such as the filename of the receptor, refer to files contained within the AutoGrow4 download.

We do not include the parameters that specify the paths to OpenBabel (*openbabel_bin_directory*), MGLTools (*mgltools_directory*), and the output (*output_dir*) directories because these paths depend on the specific computer used for testing.

```
{
	"filename_of_receptor": "/autogrow4/tutorial/PARP/4r6eA_PARP1_prepared.pdb",
	"center_x": -70.76,
	"center_y": 21.82,
	"center_z": 28.33,
	"size_x": 25.0,
	"size_y": 16.0,
	"size_z": 25.0,
	"source_compound_file": "/autogrow4/source_compounds/naphthalene_smiles.smi",
	"number_of_mutants_first_generation": 50,
	"number_of_crossovers_first_generation": 50,
	"number_of_mutants": 50,
	"number_of_crossovers": 50,
	"top_mols_to_seed_next_generation": 70,
	"number_elitism_advance_from_previous_gen": 70,
	"number_elitism_advance_from_previous_gen_first_generation": 0,
	"diversity_mols_to_seed_first_generation": 0,
	"diversity_seed_depreciation_per_gen": 0,
	"num_generations": 5,
	"number_of_processors": 12,
	"scoring_choice": "VINA",
	"LipinskiStrictFilter": true,
	"GhoseModifiedFilter": true,
	"filter_source_compounds": false,
	"start_a_new_run": true,
	"selector_choice": "Rank_Selector",
	"dock_choice": "VinaDocking", *
	"max_variants_per_compound": 1, *
	"generate_plot": false,
	"debug_mode": true,
	"reduce_files_sizes": false
}
```


Supplemental JSON 2: AutoGrow4 settings used in the benchmark experiments.

The source compounds (*naphthalene_smiles.smi*) are the same molecules from the AutoGrow 3.1.3 *directory_of_source_compounds* directory (Supplemental JSON 1), converted from PDB to SMILES. AutoGrow 3.1.3 does not separate elitism selection and seeding selection, so we set both *top_mols_to_seed_next_generation* and *number_elitism_advance_from_previous_gen* to 70 in order to match the AutoGrow 3.1.3 *top_ones_to_advance_to_next_generation* parameter. We set *GhoseModifiedFilter* to the Ghose* filter described in Table 1, to match the AutoGrow 3.1.3 Ghose filter. We also applied *LipinskiStrictFilter*, which is described in Table 1 as Lipinski*. We silenced several additional AutoGrow4 features (e.g., plotting the results, deleting temporary files, reducing file size by compression, and selecting by diversity) because these features are not available in AutoGrow 3.1.3.

An asterisk (*) is placed next to the two variables, *dock_choice* and *max_variants_per_compound*, that we varied to test different AutoGrow4 configurations. We set the *dock_choice* parameter, which specifies the docking software to use, to either *VinaDocking* or the default choice of *QuickVina2Docking*. We set the *max_variants_per_compound* parameter, which determines the maximum number of variants that the Gypsum-DL module should produce per input compound, to either 1, 3, or 5. We tested all permutations of *dock_choice* and *max_variants_per_compound*, resulting in six configurations. All unspecified AutoGrow4 parameters were set to the defaults.

The paths to the MGLTools directory (*mgltools_directory*) and the root output folder (*root_output_folder*) are again omitted from the JSON file because they depend on the specific computer used for testing.

```
{
	"filename_of_receptor": "/autogrow4/tutorial/PARP/4r6eA_PARP1_prepared.pdb",
	"center_x": -70.76,
	"center_y": 21.82,
	"center_z": 28.33,
	"size_x": 25.0,
	"size_y": 16.0,
	"size_z": 25.0,
	"source_compound_file":
	"/autogrow4/source_compounds/Fragment_MW_100_to_150.smi",
	"number_of_mutants_first_generation": 500,
	"number_of_crossovers_first_generation": 500,
	"number_elitism_advance_from_previous_gen_first_generation": 40,
	"number_of_mutants": 2500,
	"number_of_crossovers": 2500,
	"number_elitism_advance_from_previous_gen": 500,
	"top_mols_to_seed_next_generation_first_generation": 50,
	"top_mols_to_seed_next_generation": 500,
	"diversity_mols_to_seed_first_generation": 500,
	"diversity_seed_depreciation_per_gen": 5,
	"num_generations": 30,
	"number_of_processors": 280,
	"dock_choice": "QuickVina2Docking",
	"scoring_choice": "VINA",
	"selector_choice": "Rank_Selector",
	"LipinskiStrictFilter": true,
	"GhoseFilter": true,
	"PAINSFilter": true,
	"reduce_files_sizes": true,
	"max_variants_per_compound": 5,
	"filter_source_compounds": false,
	"use_docked_source_compounds": true,
	"rxn_library": "all_rxns",
	"multithread_mode": "mpi"
}
```

Supplemental JSON 3: AutoGrow4 settings used in the large-scale *de novo* experiments. The source compounds were taken from AutoGrow4's *Fragment_MW_100_to_150.smi* library, which consists of ZINC15 molecules that have molecular weights ranging from 100 Da to 150 Da. We applied the Ghose, Lipinski*, and PAINS filters. All unspecified AutoGrow4 parameters were set to the default values. The path to the MGLTools directory (*mgltools_directory*) and the root output folder (*root_output_folder*) are again omitted.

```
{
	"filename_of_receptor": "/autogrow4/tutorial/PARP/4r6eA_PARP1_prepared.pdb",
	"center_x": -70.76,
	"center_y": 21.82,
	"center_z": 28.33,
	"size_x": 25.0,
	"size_y": 16.0,
	"size_z": 25.0,
	"source_compound_file": "/autogrow4/source_compounds/PARPI_BRICS_frags.smi",
	"number_of_mutants_first_generation": 500,
	"number_of_crossovers_first_generation": 500,
	"number_elitism_advance_from_previous_gen_first_generation": 40,
	"number_of_mutants": 2500,
	"number_of_crossovers": 2500,
	"number_elitism_advance_from_previous_gen": 250,
	"top_mols_to_seed_next_generation_first_generation": 50,
	"top_mols_to_seed_next_generation": 500,
	"diversity_mols_to_seed_first_generation": 500,
	"diversity_seed_depreciation_per_gen": 25,
	"num_generations": 5,
	"number_of_processors": 280,
	"dock_choice": "QuickVina2Docking",
	"scoring_choice": "VINA",
	"selector_choice": "Rank_Selector",
	"LipinskiStrictFilter": true,
	"GhoseFilter": true,
	"PAINSFilter": true,
	"reduce_files_sizes": true,
	"docking_exhaustiveness": 25,
	"max_variants_per_compound": 5,
	"gypsum_timeout_limit": 60,
	"docking_timeout_limit": 600,
	"filter_source_compounds": false,
	"use_docked_source_compounds": true,
	"rxn_library": "all_rxns",
	"multithread_mode": "mpi",
	"start_a_new_run": true
}
```

Supplemental JSON 4: AutoGrow4 settings used in the PARPi lead-optimization experiments. The source compounds from *PARPI_BRICS_frags.smi* consist of 11 PARPi molecules and 83 PARPi-derived fragments. We generated PARPi fragments using BRICS decomposition. All unspecified AutoGrow4 parameters were set to the default values. The paths to the MGLTools directory (*mgltools_directory*) and the root output folder (*root_output_folder*) are again omitted.

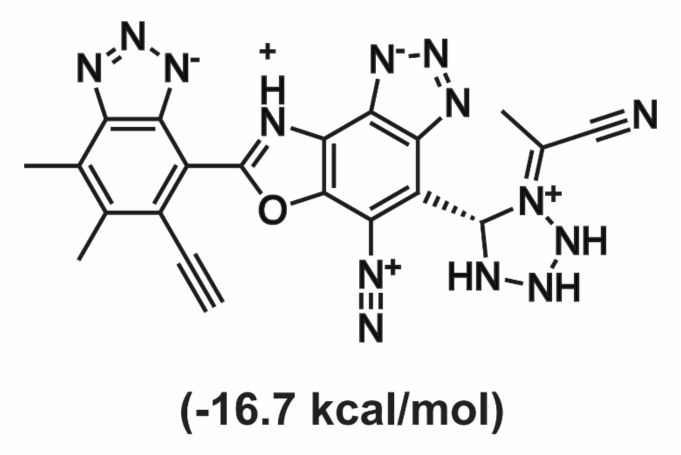

Figure S1: A high-scoring compound predicted to bind the PARP-1 catalytic domain (PDB ID: 4R6E:A), generated in the 24th generation of the large-scale *de novo* run. QVina2 was used for scoring.

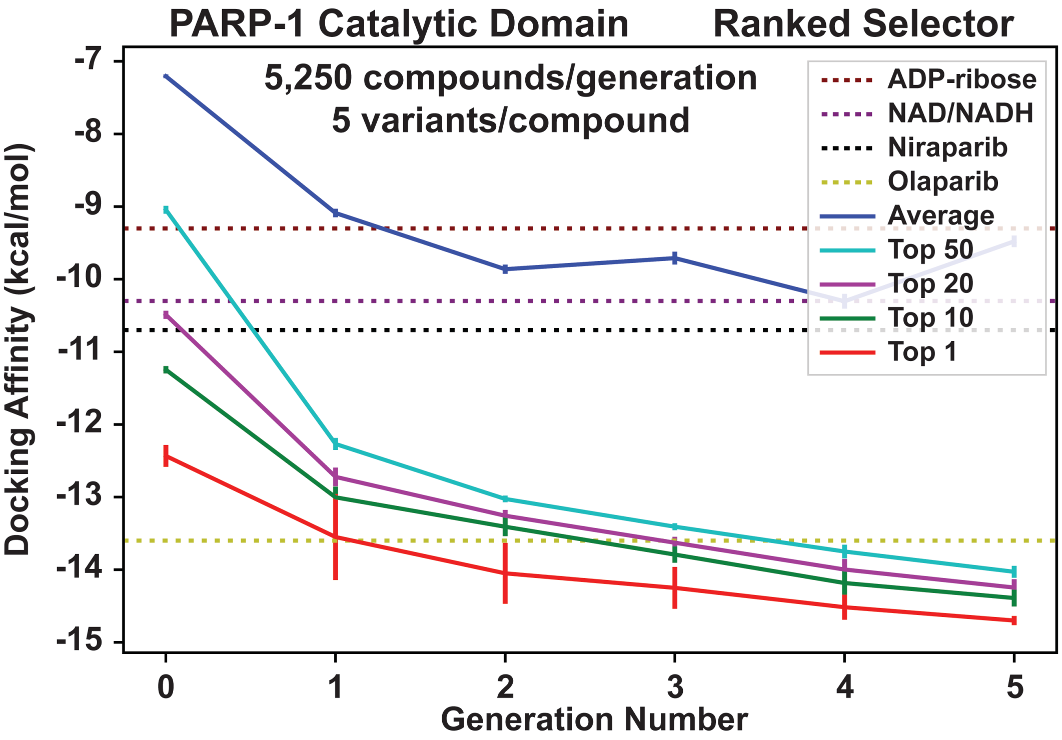

Figure S2: Results of six independent PARPi lead-optimization experiments. For each generation, the grand mean of all QVina2 scores across all six runs is shown in blue. The grand means of the top 50, 20, 10, and 1 compounds are shown in cyan, purple, green, and red, respectively. The QVina2 scores of known PARP-1 ligands are shown as dashed lines. Error bars represent standard deviations.

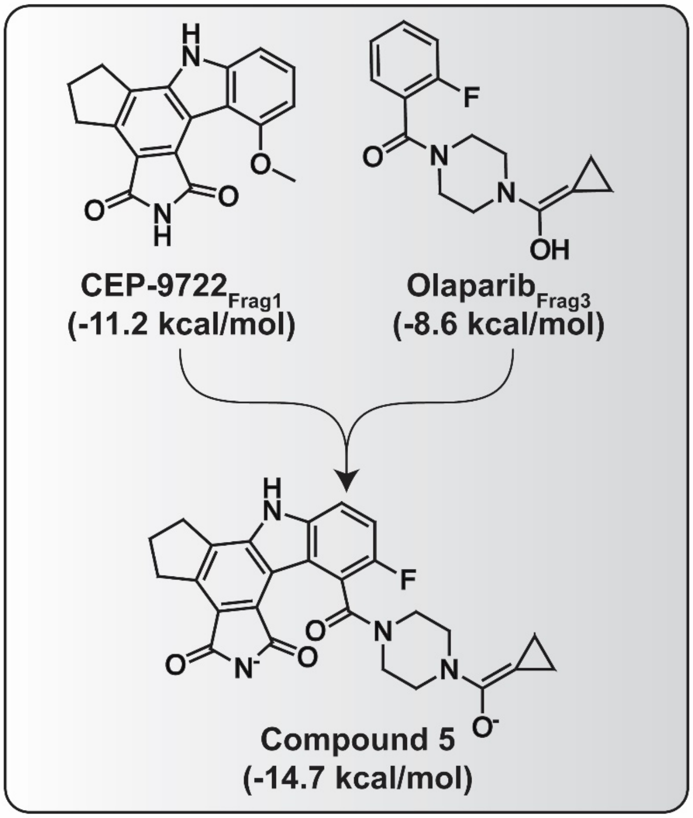

Figure S3: The lineage of Compound 5, created in the first generation of a PARPi lead-optimization run. This run was seeded with PARPi ligands and fragments. Compound 5 was one of the best compounds generated in any of the six PARPi lead-optimization runs, per the QVina2 score. It was created via a single crossover between CEP-9722_{Frag1} and $Olaparib_{Frag3}$.
